# The Relationship Between State Policies for Competitive Foods and School Nutrition Practices in the United States

**DOI:** 10.5888/pcd11.130216

**Published:** 2014-04-24

**Authors:** Caitlin L. Merlo, Emily O’Malley Olsen, Mara Galic, Nancy D. Brener

**Affiliations:** Author Affiliations: Emily O’Malley Olsen, Nancy D. Brener, Centers for Disease Control and Prevention, Atlanta, Georgia; Mara Galic, Danya Consultants, Atlanta, Georgia.

## Abstract

**Introduction:**

Most students in grades kindergarten through 12 have access to foods and beverages during the school day outside the federal school meal programs, which are called competitive foods. At the time of this study, competitive foods were subject to minimal federal nutrition standards, but states could implement additional standards. Our analysis examined the association between school nutrition practices and alignment of state policies with Institute of Medicine recommendations (IOM Standards).

**Methods:**

For this analysis we used data from the Centers for Disease Control and Prevention’s (CDC’s) report, *Competitive Foods and Beverages in US Schools: A State Policy Analysis* and CDC’s *2010 School Health Profiles* (Profiles) survey to examine descriptive associations between state policies for competitive foods and school nutrition practices.

**Results:**

Access to chocolate candy, soda pop, sports drinks, and caffeinated foods or beverages was lower in schools in states with policies more closely aligned with IOM Standards. No association was found for access to fruits or nonfried vegetables.

**Conclusion:**

Schools in states with policies more closely aligned with the IOM Standards reported reduced access to less healthful competitive foods. Encouraging more schools to follow these standards will help create healthier school environments and may help promote healthy eating among US children.

## Introduction

The prevalence of childhood obesity has tripled in the past 30 years, and more than one-third of US children and adolescents are overweight or obese (1,2). In response, researchers, physicians, policy makers, parents, and educators are seeking solutions and strategies for addressing physical activity and healthy eating in schools. US children attend school for at least 6 hours a day, 180 days a year (3). During this time, students have access to foods and beverages in multiple venues across the school campus.

Foods and beverages provided through the US Department of Agriculture’s (USDA’s) National School Lunch Program and School Breakfast Program must meet specific nutrition standards for schools to receive federal reimbursement (4,5). However, most students have access to other foods and beverages during the school day (6,7). These items, called competitive foods, tend to be high in added sugars, fat, and sodium (6,8).

Although federal legislation passed in July 2013 places greater restrictions on competitive foods in schools, at the time this study was conducted, federal standards for competitive foods only prohibited the sale of foods of minimal nutritional value anywhere school meals were served during the meal periods (9,10). However, such foods could be sold in other locations, and other foods of low nutritional value (eg, snack chips; most candy bars; noncarbonated, high-sugar beverages) could be sold anytime, anywhere on school campuses at all grade levels.

Low-nutrient, energy-dense competitive foods and sugar-sweetened beverages add 177 calories (about 8%) to students’ daily intake (6). If applied to an entire school year, these foods and beverages could contribute 31,860 calories to a student’s intake. Students who attend schools that sell low-nutrient, energy-dense foods and sugar-sweetened beverages report lower intake of fruits, vegetables, and milk at lunch; lower daily intake of fruits and vegetables; and higher daily percentages of calories from total fat and saturated fat (11–14). Students who attend schools without stores or snack bars that sell competitive foods or beverages have reduced access to sugar-sweetened beverages (15). Students also receive mixed messages when they are taught about healthful eating in class but have access to unhealthy foods and beverages on the school campus (16,17).

In response to these concerns, Congress directed the Centers for Disease Control and Prevention (CDC) to conduct a study with the Institute of Medicine (IOM) on the content of foods and beverages available on school campuses outside school meal programs and recommend standards. The result was the 2007 report *Nutrition Standards for Foods in Schools: Leading the Way toward Healthier Youth* (IOM Standards), which includes 13 science-based standards for foods and beverages available to students outside of school meal programs during and after the school day (17).

States and local education agencies can implement nutrition standards for competitive foods that exceed federal restrictions. Previous studies show that state standards can influence practices in schools (18–23). However, most studies examined this association in a limited sample or were not able to examine differences between states. Our analysis examines the extent to which school nutrition practices are associated with state policy alignment with the IOM Standards across the United States. We hypothesized that the percentage of schools reporting specific healthy nutrition practices would be higher in states that have policies that are more closely aligned with the IOM Standards.

## Methods

State policy alignment with IOM Standards has been published in *Competitive Foods and Beverages in US Schools: A State Policy Analysis *(http://www.cdc.gov/healthyyouth/nutrition/pdf/compfoodsbooklet.pdf). A description of the methods used to collect and code state policies is described in that report. For this analysis we compared state policy IOM alignment quartiles identified in that report with data on school nutrition practices from CDC’s *2010 School Health Profiles* (Profiles) surveys (24). Profiles surveys have been conducted biennially since 1996 to measure school health policies and practices. These surveys use random, systematic, equal-probability sampling strategies to produce representative samples of schools that serve students in grades 6 through 12 in each participating state, territory, large urban school district, and tribal government in the United States. Data from the 49 states with principal survey response rates from 2010 Profiles of 70% or greater and appropriate documentation were weighted to be representative of all public secondary schools in that state; these states comprised our analytic sample.

### Analysis

All analyses were descriptive and performed with SUDAAN version 10.0 software (Research Triangle Institute, Research Triangle Park, North Carolina) to account for the sampling weights. We compared each state’s overall IOM alignment quartile identified in the CDC report with the percentage of schools in each state that had nutrition practices for competitive foods measured by Profiles 2010. We also compared each state’s overall IOM alignment quartile with the percentage of schools in each state that allowed students to purchase certain snack items. Finally, we compared alignment scores for individual IOM Standard variables from the CDC report with specific Profiles survey questions.

All analyses were performed at the state level. For the Profiles surveys, each state collects data from a representative sample of schools in its jurisdiction, and CDC reports these data as the percentage of schools in that state that have a particular policy or practice in place. For our analysis, we report the median across states of the percentage of schools in each state with a particular policy or practice. For example, if 56% of schools in Alabama reported a practice, 60% of schools in Arkansas reported this practice, and 64% of schools in Alaska reported this practice, the median percentage for these 3 samples would be 60%, and the sample *n* would be 3.

We hypothesized that the median percentage of schools reporting specific healthy nutrition practices would be higher in states with policies more closely aligned with IOM Standards. We examined the median percentages in each alignment quartile category from the CDC report (none, Q1, Q2, Q3, and Q4). If the medians monotonically increased or decreased across all quartile categories, this change indicated an association. If the association was in the expected direction, we reported it as yes. If the association was not in the expected direction or there was no association, we reported it as no. In a few cases, the medians moved monotonically in the expected direction with the exception of the “none” group; this was noted as “mostly” moved in the expected direction.

## Results

As identified in the CDC report, 11 states had no policies on competitive foods (none), 19 states were in Q1, 17 states were in Q2, and 2 states were in Q3. No states had an alignment score of 75.1 or greater, so no states were in Q4.

The median proportions of schools in the states in each quartile with nutrition-related practices are shown in Table 1. For the 11 states with no state policy, the median percentage of schools that almost always or always offered fruits and vegetables at school celebrations was 28.4% (Table 1). The median percentage was 33.3% for the 19 states in Q1, 34.4% for the 17 states in Q2, and 36.2% for the 2 states in Q3. This pattern was in the expected direction.

**Table 1:** Median Percentages of Secondary Schools With Nutrition-Related Policies and Practices by Overall IOM Standards Alignment Quartile, Profiles, United States, 2010

School Nutrition Practices	School Health Profiles Survey Questions	Overall IOM Standard Alignment Quartile^a^				Expected Direction as IOM Quartile Increases	Is Pattern in Expected Direction?
		None (n = 11)	Quartile 1 (n = 19)	Quartile 2 (n = 17)	Quartile 3 (n = 2)		
Food offered at celebrations	When foods or beverages are offered at school celebrations, fruits or nonfried vegetables are always or almost always offered	28.4%	33.3%	34.4%	36.2%	Increase	Yes
Availability of snack foods or beverages in vending machines or at a school store, canteen, or snack bar	Students can purchase less nutritious food items^b^	49.1%	53.1%	33.1%	14.8%	Decrease	Mostly
	Students can purchase less nutritious beverage items^c^	69.1%	62.8%	52.8%	21.2%	Decrease	Yes
	Students can purchase foods or beverages containing caffeine	36.4%	29.0%	20.1%	5.7%	Decrease	Yes
	Students can purchase fruits (not fruit juice) or nonfried vegetables (not vegetable juice)	28.2%	33.1%	29.7%	5.3%	Increase	No
Limits serving sizes of competitive foods	School limits the package or serving size of any individual food and beverage items sold in vending machines or at the school store, canteen, or snack bar	44.8%	45.8%	47.5%	35.9%	Increase	No
Promotion of candy, fast-food, or soft drinks	Candy, meals from fast-food restaurants, or soft drinks are promoted through the distribution of products, such as T-shirts, hats, and book covers to students	2.3%	2.2%	2.7%	3.2%	Decrease	No
Prohibits advertisements for candy, fast-food restaurants, or soft drinks are in various locations at school	In the school building	53.2%	67.3%	71.8%	76.8%	Increase	Yes
	On school grounds, including on the outside of the school building, on playing fields, or on other areas of the campus	47.0%	57.1%	62.1%	67.3%	Increase	Yes
	On school buses or other vehicles used to transport students	63.7%	73.3%	74.9%	76.6%	Increase	Yes
	In school publications (eg, newsletters, newspapers, websites, or other school publications)	56.2%	60.4%	68.0%	68.3%	Increase	Yes
Prohibits all promotion	Prohibits promotion of candy, meals from fast-food restaurants, or soft drinks through the distribution of products, such as T-shirts, hats, and book covers to students and prohibits advertisements for them in the school building, on school grounds, on school buses, and in school publications	38.9%	49.1%	52.1%	57.3%	Increase	Yes

Abbreviations: IOM Standards, Recommendations in the 2007 Institute of Medicine report, *Nutrition Standards for Foods in Schools: Leading the Way toward Healthier Youth*; Profiles, CDC’s School Health Profiles, Principal Survey.

a State scores are organized into the following categories: None = states with no competitive foods policies, Quartile 1 = 0–25.0, Quartile 2 = 25.1–50.0, Quartile 3 = 50.1–75.0, and Quartile 4 = 75.1–100.0.

b Less nutritious foods include chocolate candy; other kinds of candy; salty snacks that are not low in fat (eg, regular potato chips); cookies, crackers, cakes, pastries, or other baked goods that are not low in fat; and ice cream or frozen yogurt that is not low in fat.

c Less nutritious beverages include 2% or whole milk (plain or flavored), water ices or frozen slushes that do not contain juice, soda pop or fruit drinks that are not 100% juice, and sports drinks (eg, Gatorade).

For the availability of less nutritious foods, median percentages increased from the “none” group to Q1 and then decreased in the expected direction between Q2 and Q3. Less nutritious beverages and caffeinated beverages were less available in states in the higher quartiles, as expected, but the availability of fruits and vegetables for purchase did not increase across IOM alignment quartiles as expected. Limiting package or serving size of competitive foods available for purchase increased from the “none” group to Q2, but states in Q3 had a lower median percentage of schools with this practice than the schools in Q2.

The percentage of schools that allowed the promotion of candy, fast-food restaurants, or soft drinks through the distribution of products such as T-shirts, hats, and book covers to students was low across all IOM quartiles — less than 10% of schools in any state allowed this practice, and we found no relationship between these policies and state alignment quartiles. However, the median percentage of schools that prohibited advertisements for candy, fast-food restaurants, or soft drinks in specific areas of the school campus increased across the quartiles, as did the median percentage of schools that prohibited the promotion of all competitive foods at school.

Access to chocolate candy, soda pop, sports drinks, and caffeinated foods or beverages decreased as the overall IOM alignment quartile increased (Table 2). For other kinds of candy, salty snacks, baked goods, ice cream or frozen yogurt, 2% or whole milk, and water ices, the pattern was mostly in the expected direction. Access to fruits or nonfried vegetables was not associated with overall IOM alignment quartiles. Alignment of state policies with 8 individual IOM Standard variables was generally in the expected direction (Table 3).

**Table 2:** Median Percentage of Secondary Schools in Which Students Can Purchase Snack Foods and Beverages, by Overall IOM Standards Alignment Quartile — Profiles, United States, 2010

School Health Profiles Survey Question: Students Can Purchase at School from Vending Machines or at a School Store, Canteen, or Snack Bar	Overall IOM Alignment Quartile^a^				Expected Direction as IOM Quartile Increases	Is Pattern in Expected Direction?
	None (n = 11)	Quartile 1 (n = 19)	Quartile 2 (n = 17)	Quartile 3 (n = 2)		
Chocolate candy	32.1%	29.7%	15.3%	4.6%	Decrease	Yes
Other kinds of candy	32.3%	33.4%	21.2%	8.8%	Decrease	Mostly
Salty snacks that are not low in fat	32.2%	39.3%	20.7%	12.3%	Decrease	Mostly
Baked goods that are not low in fat	32.5%	38.7%	20.4%	7.8%	Decrease	Mostly
Ice cream or frozen yogurt that is not low in fat	14.2%	24.6%	13.4%	2.9%	Decrease	Mostly
2% or whole milk (plain or flavored)	37.2%	39.0%	28.7%	9.9%	Decrease	Mostly
Water ices or slushes that do not contain juice	14.7%	17.7%	10.6%	6.8%	Decrease	Mostly
Soda pop or fruit drinks that are not 100% juice	40.1%	33.3%	21.1%	5.8%	Decrease	Yes
Sports drinks (eg, Gatorade)	63.7%	50.8%	40.5%	13.2%	Decrease	Yes
Food or beverages containing caffeine	36.4%	29.0%	20.1%	5.7%	Decrease	Yes
Fruit (not fruit juice)	27.7%	31.7%	28.3%	5.3%	Increase	No
Nonfried vegetables (not vegetable juice)	13.6%	24.9%	15.8%	2.5%	Increase	No

Abbreviations: IOM Standards = Recommendations in the 2007 Institute of Medicine report, *Nutrition Standards for Foods in Schools: Leading the Way Toward Healthier Youth;* Profiles = CDC’s School Health Profiles, Principal Survey.

a State scores are organized into the following categories: None = states with no competitive foods policies, Quartile 1 = 0–25.0, Quartile 2 = 25.1–50.0, Quartile 3 = 50.1–75.0, and Quartile 4 = 75.1–100.0.

**Table 3:** Percentage of Secondary Schools^a^ in Which Students Can Purchase Snack Foods and Beverages, by Alignment with Individual IOM Standard Alignment Category, United States, 2010

IOM Standard Description	School Health Profiles Survey Question: Students Can Purchase at School from a Vending Machine or at a School Store, Canteen, or Snack Bar	Individual IOM Standard Alignment Category^b^				Expected Direction as IOM Standard Alignment Increases	Is Pattern in Expected Direction?	
		None (n = 11)	Not Met	Partially Met	Fully Met			
Foods and beverages are caffeine free	Foods or beverages containing caffeine	36.4%	27.2% (n = 31)	16.0% (n = 6)	4.3% (n = 1)^c^	Decrease	Yes	
Foods and beverages offered during the school day are limited to Tier 1 foods: fruits and vegetables encouraged or required	Fruits (not fruit juice) or nonfried vegetables (not vegetable juice)	28.2%	32.6% (n = 25)	22.2% (n = 1)^c^	27.2% (n = 12)	Increase	No	
Foods and beverages offered during the school day are limited to Tier 1 foods: nonfat or low-fat dairy products required	Ice cream or frozen yogurt that is not low in fat	14.2%	21.6% (n = 28)	14.3% (n = 5)	5.7% (n = 5)	Decrease	Mostly	
100% fruit and vegetable juices (8 fl oz max)	Soda pop or fruit drinks that are not 100% juice	40.1%	24.4% (n = 15)	23.4% (n = 21)	15.8% (n = 2)	Decrease	Yes
Nonfat or lowfat milk	2% or whole milk (plain or flavored)	37.2%	40.4% (n = 14)	29.5% (n = 22)	14.2% (n = 2)	Decrease	Mostly
Prohibit regular (calorically sweetened) soda	Soda pop or fruit drinks that are not 100% juice	40.1%	37.3% (n = 12)	28.8% (n = 8)	18.8% (n = 18)	Decrease	Yes
Prohibit other beverages (other than soda and sports drinks) that contain added caloric sweetener	Soda pop or fruit drinks that are not 100% juice	40.1%	31.8% (n = 20)	22.4% (n = 10)	16.9% (n = 8)	Decrease	Yes
Prohibit sports drinks in the school setting	Sports drinks (eg, Gatorade)	63.7%	55.1% (n = 22)	40.5% (n = 9)	16.8% (n = 7)	Decrease	Yes

Abbreviations: IOM, the Institute of Medicine; IOM Standards, Recommendations in the 2007 Institute of Medicine report, *Nutrition Standards for Foods in Schools: Leading the Way toward Healthier Youth*; Profiles, CDC’s School Health Profiles, Principal Survey.

a Elementary school rankings were not included when determining the IOM score for these standards.

b Scores are reset for each standard and are calculated as follows: None; 0 = Not Met; 1–3 = Partially Met; 4 = Fully Met.

c Because only 1 state had this alignment score for this variable, the value reported is the percentage within that state, not the median percentage across multiple states.

## Discussion

Although several factors can influence the degree of state policy alignment with the IOM Standards, including the extent to which policy decisions are made at the local level and the level of support for obesity prevention policies in the state, our analyses generally showed that schools in states that have policies more aligned with IOM Standards reported practices that promote healthy eating more often than schools in states with policies that were less aligned or states that have no policies at all.

However, for some variables — such as allowing promotion of candy, fast-food restaurants, or soft drinks through products distributed to students — no clear relationship between state policy alignment with IOM Standards and school practices was found. This could be because the IOM Standards do not include a specific recommendation for the distribution of products promoting candy, fast-food restaurants, or soft drinks to students. Overall, few schools reported that they allow such practices, regardless of alignment quartile. It is also possible that the respondents to the Profiles questions were not aware of these forms of promotion or that these specific forms of promotion are not common.

Our results did not show a clear relationship between overall IOM alignment quartile and the median percentage of schools in each state that reported providing fruits and vegetables for students to purchase. Although we would expect schools that limit access to less healthful foods to also increase access to more healthful foods like fruits and vegetables, they may not for several reasons. Schools in states with policies that are more aligned with IOM Standards may be restricting access to all competitive foods rather than replacing less healthful foods with healthier options such as fruits and nonfried vegetables. Some schools may not provide access to fruits and vegetables because fresh produce has a shorter shelf life than prepackaged snack items. Fresh produce also may cost more to purchase and may require more work by school nutrition staff to order and stock. School officials may also be less likely to offer fruits and nonfried vegetables because they believe that students are less likely to buy them. States and school districts can provide technical assistance on strategies to promote access to and consumption of fruits and vegetables including opportunities for students to sample fruits and vegetables (25).

The results of our analysis did not show a consistent association between overall IOM alignment quartiles and the median percentage of schools in each state that reported limiting package or serving size of competitive foods available for purchase. The IOM Standards do not address limiting the package size of snack foods specifically. However, implementing IOM Standard 3 (limits on calories per portion as packaged) would in effect reduce the allowable package size of many snack foods. Because school officials who answer the Profiles surveys are unlikely to view these practices as similar, limiting package or serving size of competitive foods may have been underreported.

When we examined the availability of individual snack foods and beverages by overall IOM alignment quartiles (Table 2), the association between state policy alignment and school practices was in the expected direction for 4 of the 12 items. For 6 items, the pattern was mostly in the expected direction, but the percentage of schools reporting that students could purchase certain foods was actually lower in states with no state policies on competitive foods than in states in Q1. This may in part reflect that several of the states in Q1 had policy recommendations, but not requirements, for nutrition standards and were actually closer in practice to states without policies.

The observed associations between state policies and school practices in the expected direction were a positive indication that some aspects of state policies have been implemented in schools. However, we identified some healthy nutrition practices that were less likely to be implemented, and more effort could be made to support school practices that align with IOM Standards. For example, even in the third quartile (Q3), almost half of schools did not report prohibiting all forms of promotion of less nutritious foods. This is concerning because it has been shown that advertising influences the purchase requests, preferences, and diets of children (16). More than $149 million is spent on marketing of foods and beverages in schools annually, with carbonated beverages and noncarbonated beverages making up the majority of in-school marketing expenditures (26). Schools can create an environment with consistent messages to students about healthful eating by implementing policies and practices that only allow marketing of foods and beverages that align with nutrition standards (25,26). Our analysis indicated that only 3 states had policies that addressed food and beverage marketing on school campuses. States that do not have policies on marketing and promotion of less healthful foods in schools could consider adopting such policies as a way to help reinforce messages to students about healthy dietary choices.

Our analysis did not find that access to fruits or nonfried vegetables (excluding fruit juice and vegetable juice) increased with overall IOM alignment quartiles. This could have been a result of state policies that emphasize which foods and beverages should not be available in schools but do not specify which items should be available. States could revise their policies to provide specific guidance to schools on how to increase students’ access to healthful foods and beverages. Additionally, most state policies use nutrient-based standards, which place limits on the amount of certain nutrients (eg, calories, fat, sodium, sugar) in foods offered, rather than food-based standards, which identify specific food groups to allow (eg, fruits, whole grains) or not allow (eg, caffeinated beverages). Nutrient-based standards may be easier for schools to implement because they use information that is readily available on the Nutrition Facts labels. However, food-based standards may be more useful for identifying food groups that schools can offer to better align with national *Dietary Guidelines for Americans* recommendations (27). The new USDA standards for competitive foods use a combination of food-based and nutrient-based standards (28), which may help schools identify healthier options to make available to students.

This study had several limitations. First, the Profiles survey questions do not completely align with the IOM Standards. Most of the IOM Standards apply to the school day, and only 2 apply to after the school day. The Profiles questions do not indicate a time frame, so we do not know if respondents considered only the school day in their responses. We could better understand the association between state policies and school practices if the Profiles questions included specific time frames.

Second, the Profiles data apply only to public secondary schools and do not reflect practices at private schools or elementary schools, potentially affecting the generalizability of our results. However, the exclusion of private schools from the Profiles data is unlikely to have affected the results of our analysis because state policies do not always apply to private schools. The state policy variables used to create the overall IOM alignment quartiles scores in our analysis include elementary school policies. Although state policies for competitive foods tend to be more aligned with IOM Standards at the elementary school level than at middle and high school levels, the results of our analysis show that practices in secondary schools tend to be associated with overall IOM alignment quartiles even when elementary school policies are included in the overall policy alignment scores (Table 1, Table 2).

Third, Profiles data were self-reported by principals or their designees, and the accuracy of their responses to the Profiles questions regarding the food products being sold or advertised in their schools was not verified by other sources. Additionally, our analysis looks only at state policies and does not account for policy changes at local levels that may influence school practices. All school districts that participate in federal school meal programs are required to have a local school wellness policy that includes nutrition standards for all foods available during the school day (29). We did not control for local policy alignment with IOM Standards and therefore cannot determine if state policies are responsible for the associations we identified.

Lastly, this analysis does not account for the amount of time that a state policy has been in place. Future research could examine how long a policy has been in place and whether a policy has an enforcement mechanism. Future research also could use a nationally representative sample, such as CDC’s School Health Policies and Programs Study (30). Additionally, longitudinal studies could examine whether state policies affect student dietary behavior.

Although our analysis shows that associations between school nutrition practices and alignment of state policies with IOM Standards were generally in the expected direction, states could take additional steps to promote a healthy school nutrition environment. In addition to clearly defining which foods and beverages are not allowed in schools, state policies could define the types of foods and beverages schools could offer in place of less healthful options on the basis of the IOM’s definition of recommended foods or the new USDA standards for competitive foods and beverages. States could consider addressing marketing and promotion of foods and beverages in policies and include mechanisms for monitoring and enforcement to help ensure that policies are implemented at the school level. States can provide technical assistance to schools and school districts on implementing existing policies for competitive foods including the new USDA standards. Additionally, states can continue to revise existing policies to meet or exceed the new USDA standards.
